# Sinonasal Biomarkers Defining Type 2-High and Type 2-Low Inflammation in Chronic Rhinosinusitis with Nasal Polyps

**DOI:** 10.3390/jpm12081251

**Published:** 2022-07-29

**Authors:** Eugenio De Corso, Silvia Baroni, Stefano Settimi, Maria Elisabetta Onori, Rodolfo Francesco Mastrapasqua, Eliana Troiani, Giacomo Moretti, Donatella Lucchetti, Marco Corbò, Claudio Montuori, Alessandro Cantiani, Davide Paolo Porru, Simone Lo Verde, Giuseppe Alberto Di Bella, Cristiano Caruso, Jacopo Galli

**Affiliations:** 1 Unit of Otorhinolaryngology and Head-Neck Surgery, Fondazione Policlinico A. Gemelli IRCCS, 00168 Rome, Italy; eugenio.decorso@policlinicogemelli.it (E.D.C.); jacopo.galli@unicatt.it (J.G.); 2 Unit of Chemistry, Biochemistry and Molecular Biology, Fondazione Policlinico A. Gemelli IRCCS, 00168 Rome, Italy; silvia.baroni@policlinicogemelli.it; 3 Department of Basic Biotechnological Sciences, Intensive Care and Perioperative Clinics, Università Cattolica del Sacro Cuore, 00168 Rome, Italy; mariaelisabetta.onori@guest.policlinicogemelli.it (M.E.O.); eliana.troiani@unicatt.it (E.T.); giacomo.moretti@policlinicogemelli.it (G.M.); 4 Department of Head-Neck and Sensory Organs, Università Cattolica del Sacro Cuore, 00168 Rome, Italy; rodolfomastrapasqua@gmail.com (R.F.M.); marco.corbo@icloud.com (M.C.); claudio_montuori@libero.it (C.M.); cantiani.ale@outlook.it (A.C.); davide.cp@tiscali.it (D.P.P.); loverde.simo@gmail.com (S.L.V.); giuseppealberto.dibella@gmail.com (G.A.D.B.); 5 Department of Translational Medicine and Surgery, Università Cattolica del Sacro Cuore, 00168 Rome, Italy; donatella.lucchetti@unicatt.it; 6 Department of Medical and Surgical Sciences, Digestive Disease Center, Fondazione Policlinico A. Gemelli IRCCS, 00168 Rome, Italy; cristiano.caruso@policlinicogemelli.it

**Keywords:** chronic rhinosinusitis, eosinophilic rhinitis, nasal polyposis, biomarker, medical therapy of chronic rhinosinusitis, disease severity

## Abstract

The complex pathophysiology of chronic rhinosinusitis with nasal polyps (CRSwNP) generates a spectrum of phenotypes with a wide variety of inflammatory states. We enrolled 44 very-likely-to-be type 2 CRSwNP patients in order to evaluate the load of inflammation and to analyze human interleukins in nasal secretion. Clinical data were collected to evaluate the severity of the disease. High levels of IL-5, IL-4, IL-6, and IL-33 were detected in all type 2 CRSwNP patients. By analyzing type 2 cytokine profiles and local eosinophil count, we identified two coherent clusters: the first was characterized by high levels of IL-4, IL-5, IL-6, and a high-grade eosinophil count (type 2-high); the second had lower levels of cytokines and poor or absent eosinophilic inflammation (type-2 low). IL-5 levels were significantly higher within the type 2 cytokine and it was the most reliable biomarker for differentiating the two clusters. In type 2-high inflammatory profile clinical scores, the mean number of previous surgeries and need for systemic corticosteroids were significantly higher compared to type 2-low. Our research demonstrated the potential role of type 2 biomarkers, and in particular, of IL-5 in identifying patients with a more severe phenotype based on a high inflammatory load.

## 1. Introduction

Chronic rhinosinusitis with nasal polyps (CRSwNP) is a complex disorder characterized by different phenotypes and endotypes; chronic sinonasal mucosal inflammation is the pathophysiologic mainstay of the disease, and it is caused by different pathogenic mechanisms [1]. Three kinds of cell-mediated immune responses (type 1, type 2, and type 3) can be highlighted and may explain the heterogeneity of CRSwNP by affecting the composition of the granulocytic airway infiltrate [2]. The complex pathophysiology may generate a spectrum of different severities and is associated with a wide variety of inflammatory states [3].

In Western countries, CRSwNP predominantly manifests eosinophil-dominant type 2 inflammation involving various cytokines (e.g., IL-4, IL-5, IL-13, and IL-33) that regulate the proliferation and differentiation of eosinophils, thereby affecting their transmigration and enhanced survival in the peripheral sinonasal mucosa [4]. Type 2 inflammation has been extensively studied in sinonasal mucosa and has been associated with poorer disease control (recurrent diseased mucosa and worse symptoms) compared to non-type 2 disease [5,6,7,8,9]. Consequently, these patients may need repeated surgeries and/or longer and stronger dosages of medical interventions, including local and systemic steroids and/or targeted biologics [10].

There is a spectrum of severity in CRS patients, and recurring uncontrolled status is certainly the major obstacle in successfully treating type 2 CRSwNP, leading to a substantial burden for patients and the healthcare system [1,10]. A stratification of clinical severity is needed to modulate medical therapy and to focus on potential candidates for personalized targeted therapy [11]. Several biomarkers related to type 2 inflammation have been suggested as potential predictors of severity and poor outcome with treatments [12,13,14]. In fact, studies have revealed that the underlying immunopathologic characteristics of inflammation may determine the differences in the clinical features based on the endotype dominance and grade of inflammation, defined as the “inflammatory load” [14,15,16]. Accordingly, in our previous experience we demonstrated that preoperatively defining the type of inflammation and the inflammatory load may predict poor control in CRSwNP patients treated by surgery (ESS) and long-term local corticosteroids [17].

The objective of this study was to verify the hypothesis that a different subtype of type 2 CRSwNP patients may be identified based on the specific inflammatory load; type 2 inflammation was defined according to criteria defined by the European Position Paper on Rhinosinusitis and Nasal Polyps (EPOS 2020) [1]. The latter was measured by investigating levels of the most representative type 2 interleukins (IL)s in nasal secretion of enrolled patients. The first endpoint of the study was to determine which biomarkers in nasal secretion may be useful to measure the different loads of type 2 inflammation (high or low). The second objective was to understand if a different sub population of type 2 CRSwNP could be identified based on a different inflammatory load, and if they were characterized by a different severity of the disease.

## 2. Materials and Methods

### 2.1. Study Design and Study Population

This was an analytical observational study (level of evidence: 3) performed at the Rhinology Unit, “A. Gemelli Hospital Foundation IRCCS”, Catholic University School of Medicine and Surgery, Rome, Italy. Patients referred to our Rhinology unit were screened and enrolled in the period between October 2020 and December 2021. Based on inclusion and exclusion criteria, we included 44 patients with primary very-likely-to-be type 2 diffuse CRSwNP (16/44 females; mean age: 44.3 years old; age range 28–76). The study was approved by our institutional ethical committee (protocol n.11177/20; ID number: 3036). All methods were performed in accordance with the relevant guidelines and regulations. Table 1 presents the demographics and phenotyping of the overall population.

The inclusion criteria were:

- Male or female, 18–65 years;
- Primary diffuse CRSwNP defined according to EPOS 2020 criteria [1];
- Very high probability of type 2 inflammation based on one of these EPOS criteria: blood eosinophils >250 cells per microliter or blood total IgE >100 UI/L or local eosinophilia in the sinonasal mucosa with at least a mean of 10 cells/hpf at microscopic observation;
- Patients with significant nasal symptoms with a sinonasal outcome test (SNOT-22) >20 [18];
- Patients who had performed an additional work-up in the last 3 months including evaluation by a pulmonologist or allergologist, maxillo facial CT scan, eosinophil blood count;
- Approval by the investigators as a suitable subject for participation in the study without breach of safety or risk to the patient;
- Willingness and ability to provide written informed consent.
- The exclusion criteria were:
- CRSwNP previously treated or in ongoing treatment with biologics.
- Acute exacerbation of chronic rhinosinusitis as defined in the EPOS 2020 guidelines [1];
- Primary localized CRS;
- Secondary diffuse or localized CRS (cystic fibrosis, sinonasal tumors, primary ciliary dyskinesia, or autoimmune disease);
- Local or systemic steroid treatment less than 3 months before inclusion in the study, continuous systemic steroid treatment;
- Patients with a history of nasal surgery like septoplasty, cosmetic surgery, and active smokers;
- Current malignancy of any kind, previous radiotherapy for head or neck cancer.

### 2.2. Study Protocol

Patients were evaluated at the screening visit (V0) and one months later (V1).

At V0, all patients underwent a baseline interview including information on blood eosinophilia, asthma (based on evaluation by pulmonologists), hypersensitivity to aspirin (based on a reported history of adverse reactions associated with aspirin and/or other nonsteroidal anti-inflammatory drugs or on allergological evaluation). Data and images from a recently performed CT were collected. We gathered information about disease control including previous surgery and previous brief cycles of steroids (cycles with >5 and <21 days of systemic corticosteroids, in the last year), previous surgery, previous medical treatment, previous cycle of systemic corticosteroids (we considered the number of cycles in the last year). Patients willing to participate in the study were required to stop medical therapy for 4 weeks before V1. All subjects signed a written informed consent.

At V1, all patients underwent:

- Symptom score analysis by specific questionnaires. In particular, we used the validated Italian version of SNOT-22 (possible total score range: 0–110. A SNOT-22 score <20 is suggestive of mild symptoms) and a visual analog scale of 0–10 to evaluate impaired sense of smell and nasal obstruction. For each symptom, patients had to indicate, using a scale ranging from 0 to 10, their answer to the question: “How troublesome is your symptom”? Patients were told that 0 indicated “absence of symptom” and 10 indicated “symptom is extremely bothersome” [18].
- Nasal endoscopy findings were noted using the Lund–Kennedy Endoscopic Score (LKES) [19] to assess the following parameters: nasal mucosa edema (absent = 0; mild-moderate = 1, polypoid degeneration = 2), presence of secretion (absent = 0, hyaline = 1, thick and/or mucopurulent = 2) and presence of polyps (absent = 0, limited to the middle meatus = 1, extended to the nasal cavity = 2), scarring (absent = 0; mild = 1; severe = 2), and crusting (absent = 0; mild = 1; severe = 2). The assessment was performed bilaterally, a combined score (right + left side) of 0–20 was possible.
- Nasal cytology to determine the presence and the type of sinonasal inflammation [20]. The examination was carried out on the material taken from the lower and middle turbinate bilaterally, by “scraping” the mucosa with a Rhino-probe (Farmark SNC, Milan, Italy). Samples were mounted on a slide, immediately fixed with 95% ethanol, and stained with the May–Grunwald–Giemsa method. Eosinophil and neutrophil counts were expressed as mean value per high power field (hpf) on at least 10 of the richest observed fields at high magnification (×400). The percentage of eosinophils and neutrophils on total cellularity was also provided.
- Nasal secretions were collected bilaterally from each patient [21]. A scissored postoperative sinus sponge pack Merocel (Medtronic Xomed, Inc., Jacksonville, FL, USA) was introduced parallel to the sagittal plane into the middle meatus of each nostril. Thereafter, 1 mL of 0.9% normal saline was added to the sponge to extract the secretion. Each sponge was maintained for 5 min, transferred to a 5 mL syringe (BD, Franklin Lakes, NJ, USA), and centrifuged at 1500× *g* for 15 min at 4 °C. The supernatants were divided in aliquots and stored at –80 °C until further analysis.

### 2.3. Biochemical Assay

For the detection of interleukins in nasal secretions, a multiplex ELISA-based immunoassay was used (Luminex xMAP system, Bio-Plex^®^ 200 System Bio-Rad Laboratories, Hercules, CA, USA) as previously reported [13]. We used the Human Magnetic Luminex^®^ Screening Assay (BioTechne, Milan, Italy), which allows the simultaneous detection of the following circulating analytes: IL-5 [limit of detection (LOD): 0.13 pg/mL], IL-22 (LOD 1.90 pg/mL), IL-33 (LOD 0.50 pg/mL), IL-17A (LOD 0.90 pg/mL), IL-13 (LOD 1.50 pg/mL), GM-CSF (granulocyte-macrophage colony-stimulating factor; LOD 0.50 pg/mL), IL-6 (LOD 0.15 pg/mL), IFN-γ (Interferon-Gamma; LOD 060 pg/mL).The assay was performed according to the manufacturer’s instructions and Bio-Plex Manager Software (Bio-Rad Laboratories, Hercules, CA, USA) was used for data analysis. The IL-4 test is not provided by the manufacturers of the Luminex bioplex assay and for this reason it was performed using the Quantikine^®^ Human IL-4 Immunoassay (R&D systems^®^, MN, USA) (LOD 0.125 pg/mL).

### 2.4. Statistical Analysis

Statistical analysis was performed using SPSS 25 for Windows (IBM Corp. Released 2017. IBM SPSS Statistics for Windows, Version 25.0. IBM Corp: Armonk, NY, USA). Continuous values, such as levels of interleukins, symptom scores, endoscopic scores, and cell counts were expressed as mean ± standard deviation (SD). We performed a 2-step cluster analysis model with a maximum of seven ramifications that identified two main coherent clusters. Comparisons between measures of the two groups were performed with the Mann–Whitney U-test for non-normally distributed data and *t*-test for paired samples in normally distributed values. The Chi square test was used to compare categorical data such as prevalence and incidence. The strength of the correlation between the two parameters was obtained by Spearman’s rank correlation test. The results were considered significant for *p* values < 0.05. We performed receiver operator characteristic (ROC) curve analysis for IL-4, IL-5, IL-6, IL-33. The ROC curve is a regression model that estimates specificity and sensibility for every possible cut-off in the variable value range. Goodness of fit for different ROC curves can be compared using area under the curve (AUC) analysis for single coordinates of specificity and sensibility. Although AUC gives an estimate of general goodness of fit for the different models, the choice of the cut-off for the single model must be calibrated based on clinical purposes while balancing the need to minimize false negatives or positives.

## 3. Results

### 3.1. Magnetic Luminex^®^ Multiple Assays

**Figure 1:**
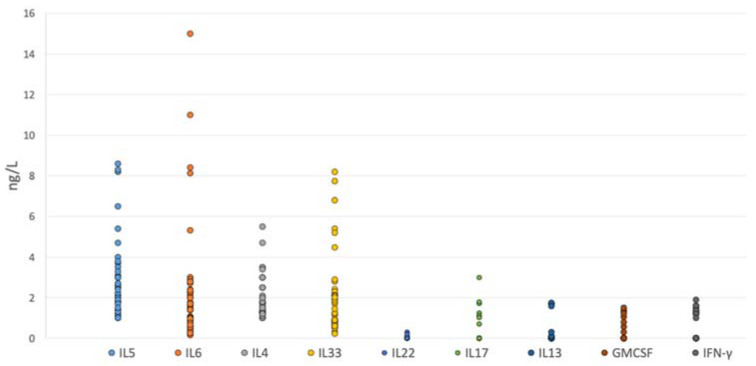
Mean levels of cytokines. Histogram showing the mean levels of cytokines in the overall population, measured in ng/L.

By Luminex assay, we detected high levels of IL-6 (3.1 ± 2.1 ng/L), IL-5 (2.8 ± 0.9 ng/L), IL-4 (1.9 ± 0.9 ng/L) and IL-33 (1.9 ± 0.9 ng/L). On the other hand, very low levels of IFN-γ (0.4 ± 0.2 ng/L), IL-13 (0.04 ± 0.2 ng/L), IL-17 (0.3 ± 0.2 ng/L), and GM-CSF (0.2 ± 0.2 ng/L) were noted. Nevertheless, all samples showed a detectable interleukin level that was within the sensitivity range provided by the manufacturer for each specific test. Figure 1 summarizes the levels of cytokines detected by the Luminex assay.

The differences between the levels of IL-5, IL-6, IL-4, and IL-33 levels were each significantly higher compared to IL-13, IL-17, IL-22, IFN-γ, and GM-CSF (*p* < 0.05). Finally, taking into consideration only type 2 specific cytokines, we observed that levels of IL-5 were significantly higher compared to IL-4 and IL-33 and that the difference was statistically significant (*p* < 0.01).

**Figure 2:**
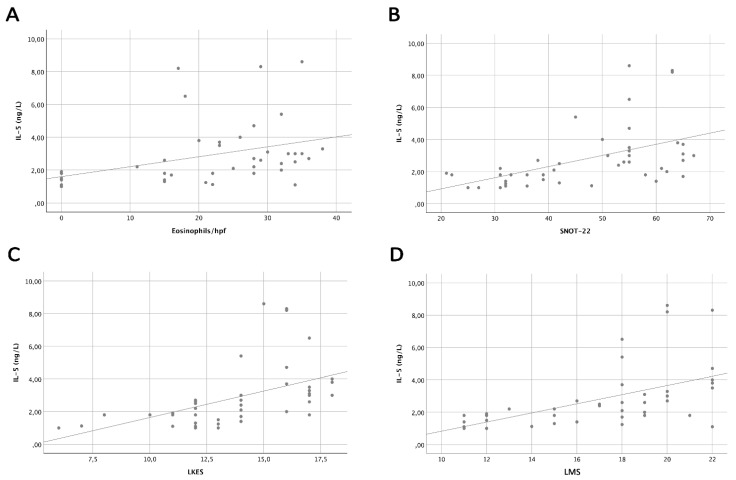
Correlation between IL-5 and main clinical parameters. Scatter plots reporting the results of Spearman’s rank test, with a significant linear correlation between IL-5 levels and eosinophilic count/HPF (**A**), SNOT-22 score (**B**), LKES score (**C**), LMS score (**D**). Abbreviations: hpf = high power field.

After correlating the levels of the most relevant cytokines with clinical parameters of all enrolled patients, we found that IL-5 had the best correlation with eosinophilic count and clinical scores. Specifically, based on the Spearman’s rank test there was a significant linear correlation between IL-5 levels and eosinophilic count/hpf (rs: 0.569; *p* < 0.01), SNOT-22 score (rs: 0.605; *p* < 0.01), Lund–Mackey score (LMS) (rs: 0.664; *p* < 0.01), and Lund–Kennedy endoscopic score (LKES) (rs: 0.670; *p* < 0.01) (Figure 2).

### 3.2. Cluster Analyses

**Figure 3:**
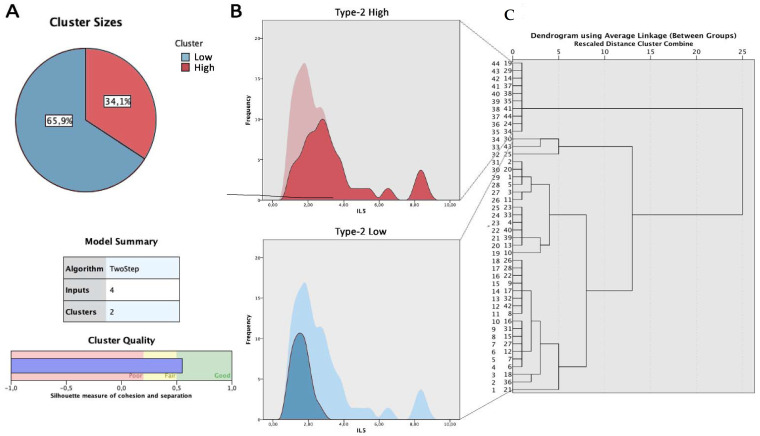
Results of cluster analysis. (Panel (**A**)): cluster sizes and model summary, displaying a good model quality. (Panel (**B**)): Cell distribution with frequency of IL-5 in type 2-high subgroup (red graphic) and in type 2-low subgroup (blue graphic). (Panel (**C**)): Dendogram of clusterization obtained using average linkage between the two clusters.

We obtained the best cluster analyses model using cytokine levels of IL-5, IL-6, and IL-4, and local eosinophil count. We therefore identified two main clusters of patients that differed from each other in the load of inflammation (Figure 3). In the first cluster, which we called type 2-high (Group 1: n = 15 patients), a higher level of IL-5, IL-6, IL-33, and IL-4 was detected compared to the type 2-low group (Group 2: n = 29 patients). In particular, mean IL-5 levels were significantly higher in the type 2-high than in type 2-low (3.45 ± 2.0 ng/L vs. 1.57 ± 0.4 ng/L) (*p* < 0.01).

A significant difference between the two groups was observed in mean IL-6 levels (4.1 ± 3.3 ng/L vs. 1.3 ± 0.9 ng/L (*p* < 0.05)) and mean IL-4 levels (2.2 ± 1.1 ng/L vs. 1.3 ± 0.2 ng/L (*p* < 0.05)). Finally, we found a difference in IL-33 levels between the two groups, although this did not reach statistical significance (2.2 ± 2.1 ng/L vs. 1.5 ± 1.4 ng/L (*p* > 0.05)).

**Figure 4:**
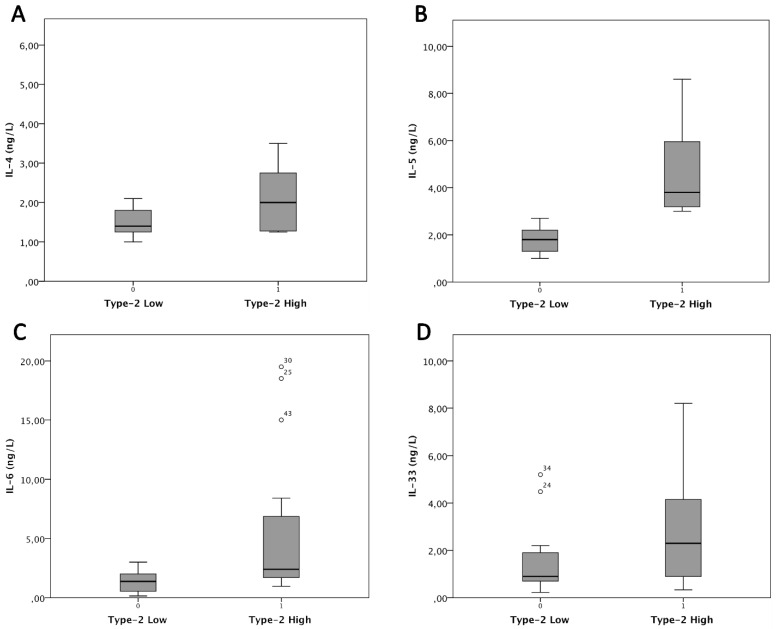
Cytokines’ levels based on inflammatory load. Box plots report the mean levels of the most representative cytokines according to the load of type 2 inflammation (high and low). Differences in mean levels of IL-4 (panel (**A**)), IL-5 (panel (**B**)), and IL-6 (panel (**C**)) were significant between the two subgroups, while IL-33 (panel (**D**)) showed a non-significant difference.

Results comparing the cytokine levels between the two subgroups are summarized in Table 2 and shown in Figure 4.

**Figure 5:**
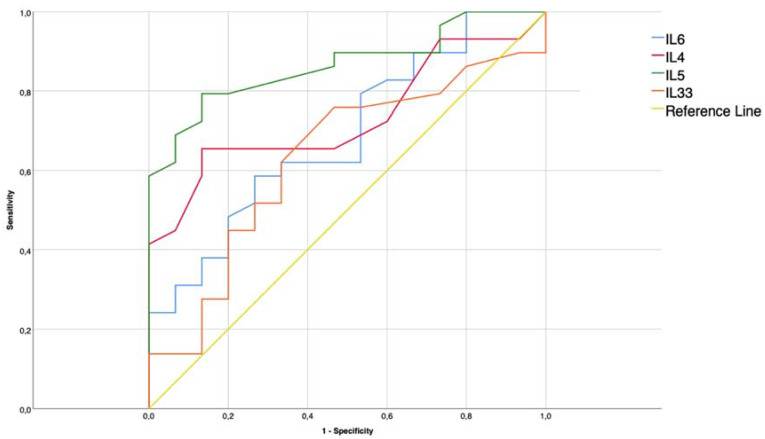
Results of ROC Analysis. ROC curves generated for IL-5, IL-4 IL-6, and IL-33 and reference line for AUC 0.5 shown for comparison. Marker on the IL-5 curve corresponds to the cut-off value of 2.3 ng/L at the coordinates of 93.3% sensibility and 70% specificity.

In ROC analyses, IL-5 was the best biomarker that could identify the two clusters among the cytokines detected in nasal secretions. In Figure 5, the ROC curves for IL-5, IL-4 IL-6 and IL-33 are shown. Specific ROC analyses for IL-5 levels in nasal secretions revealed that the area under the curve (AUC) was 0.866 (95% CI 5 0.761–0.970), a sensitivity of 70%, and a specificity of 93.3% were obtained for a cut-off of IL-5 superior to 2.3 ng/L.

Finally, a significant difference in local eosinophilia was observed between group 1 and group 2 with a mean eosinophil count per high power field (Ec-hpf) of 27.8 ± 6.1 and 4.7 ± 2.5 (*p* < 0.05), respectively. In ROC analyses, we observed that the AUC was 0.966 (95% CI 5 0.957–1.000) and an eosinophil count >15.5 Ec-hpf was highly predictive for the type 2-high cluster.

### 3.3. Clinical Characteristics of Type 2-High and Type 2-Low Phenotypes

A clinical evaluation of the two subgroups of patients (type 2-high and type 2-low) was performed (Table 3).

We observed that the number of patients with blood eosinophilia >250/μL was significantly higher in group 1 compared to group 2: 8/15 (53.3%) vs. 6/29 (20.6%) (*p* < 0.01), respectively. In analyzing the comorbidities, we observed that the proportion of asthmatic patients needing regular local therapy was higher in group 1 compared to group 2 (10/15 (66.6%) vs. 15/29 (51.17%) (*p* > 0.05)) although the difference was not significant. We did not find a difference in the allergological profile of patients. Allergy was detected in 7/15 (46.6%) in group 1 and in 13/29 (44.8%) (*p* > 0.05)] in group 2 (*p* >0.05). Finally, the prevalence of NSAID-exacerbated respiratory disease (NERD) hypersensitivity was similar in the two groups: 3/15 (20%) in the first group and 6/29 (20.6%) in the second groups.

A specific analysis was performed on scores of the extension and severity of disease in order to elucidate a different clinical burden of disease. Mean SNOT 22 and LKES scores were significantly higher in type 2-high compared to type 2-low: 54.1 ± 10.1 vs. 42.7 ± 13.8 (*p* < 0.01) and 14.9 ± 2.5 vs. 12.8 ± 3.0 (*p* < 0.01), respectively. The mean VAS impaired sense of smell and VAS nasal obstruction were significantly higher in group 1 than in group 2: 8.8 ± 1.7 vs. 7.0 ± 2.3 (*p* < 0.01) and 8.5 ± 1.5 vs. 7.0 ± 1.9 (*p* < 0.01), respectively. Finally, the mean Lund–Mackay score was significantly higher in group 1 compared to group 2: 19.8 ± 1.6 vs. 15.2 ± 3.4 (*p* < 0.01), respectively.

A significant difference was observed in previous disease control, and the mean number of previous surgeries was significantly higher in group 1 than in group 2 (2.3 ± 1.1 vs. 1.5 ± 1.1) (*p* < 0.05). In addition, the proportion of patients who had undergone more than three brief cycles of systemic corticosteroids in the last year was higher in the group 1 compared to group 2: 10 of 15 patients (66.6%) in group 1 vs. 8 of 30 (26.6%) in group 2 and the difference was statistically significant (*p* < 0.01).

## 4. Discussion

The objective of this study was to verify the hypothesis that different subtype of type 2 CRSwNP patients may be identified based on the specific inflammatory load. From a clinical point of view, a stratification of clinical severity may be very useful in routine clinical practice in order to identify patients with a poor chance of achieving control with surgery, to modulate medical therapy, and mainly, to focus on potential candidates for personalized targeted therapy. For all these reasons, we focused our attention on type 2 CRSwNP patients because they are actually the best candidates to target therapy because they have high risk of poor disease control, may present several recurrences after surgery, have poor quality of life and represent a burden for the healthcare system.

In the current study, we focused on the assessment of biomarkers in nasal secretions of very-likely-to-be type 2 CRSwNP patients based on EPOS criteria, to try to verify the hypothesis that sub-groups of patients may be identified based on the inflammatory load. We used the Luminex technique, which was designed to provide accurate, highly sensitive, and reproducible results for multiple biomarkers simultaneously with sensitivity that is similar to ELISA. Higher mean levels of IL-5, IL-4, IL-6, and IL-33 were detected in the nasal secretions of all enrolled CRSwNP patients compared to levels of IL-17, IFN-γ, IL-22, IL-13, and GM-CSF, which were seen at a very low level. Among the type 2 specific inflammatory cytokines, we observed that IL-5 was the most easily detected and its levels were significantly higher compared to IL-4 and IL-33; furthermore, it exhibited the best correlation with nasal eosinophil count and clinical parameters of CRS severity such as SNOT-22, LKES, LMS.

Few data are actually available regarding the measurement of locally secreted biomarkers and our results seem to indicate that it could be an easy and rapid diagnostic tool, compared to the more invasive tissue sampling. Our data suggested a potential role of IL-5 in nasal secretion as a reliable indicator of the type-2 inflammatory load and of the severity of the disease. Our results agree with a previous report that IL-5 is a key cytokine in nasal polyposis and it is strongly associated with eosinophilia [22,23]. The important role of IL-5 in CRS pathophysiology was also confirmed by Tomassen et al. [24], who demonstrated that patients with higher levels of IL-5 expression exhibited the highest prevalence of nasal polyposis and asthma. In contrast, the group of patients with lower IL-5 expression consisted mainly of patients with CRSsNP.

We performed cluster analysis including levels of type 2 cytokines (IL-5, IL-4) and IL-6, which is not a specific type 2 cytokine, but is considered an upstream inflammatory cytokine that reflects the grade of inflammation [25]. Among specific type 2 cytokines in nasal secretions, IL-5 was a reliable biomarker that could differentiate clusters with high and low inflammatory load. Moreover, we analyzed the clinical features of the two sub-groups of patients based on sinonasal inflammatory load, observing that clinical scores of disease severity such as LMS, LKES, and SNOT-22 scores were significantly higher in type 2-high inflammatory profiles. In addition, we concluded that patients with higher IL-5 levels in nasal secretions may be classified as type 2-high, which is associated with a more severe disease and requires more integrated care to obtain adequate disease control using oral corticosteroids. These patients, despite maximal medical therapy and surgery, have a high risk of remaining recalcitrant and biologics should be taken into considerations to optimize treatment. Several authors have demonstrated that high eosinophilic infiltration and high IL-5 expression in CRSwNP correlates with a higher rate of polyp recurrence [17,18,19,20,21,22,23,24,25,26]. Nevertheless, to the best of our knowledge this is the first study showing that two different phenotypes of type 2 CRSwNP may be identified based on the load of inflammation.

With regard to the other cytokines, IL-4 and IL-6 were significantly higher for the type 2-high group compared to type 2 -ow one. Furthermore, IL-33 mean values were higher in the type 2-high group, but the difference was not statistically significative, probably due to a higher variability among patients, thus resulting in a high standard deviation. A comment should also be made about the other cytokines that we included in our analysis due to their importance in the pathophysiology of type 2 inflammation, namely, IL-13 and GM-CSF [7]. We note that the low levels observed are not indicative of their low importance in the inflammatory process, but it should be emphasized that they cannot be considered reliable biomarkers for diagnostic purposes. In this regard, we should consider that for these specific cytokines the low levels in nasal secretion and the limits of the detection methods that we adopted, might have influenced our results.

Some authors [27,28] have suggested that eosinophil counts and/or percentage in peripheral blood might be a biomarker for severe intractable cases; in agreement with this possibility, we observed that the prevalence of patients with blood eosinophilia was significantly higher in the group with a type 2-high inflammatory profile. On the other hand, the topic of local nasal eosinophilia is still controversial considering that a large fluctuation in eosinophil number may be observed in nasal polyps, with many unpredictable and regional asymmetries as well as significant geographic and ethnic differences [1,29]. It has been demonstrated that tissue eosinophilia correlates with extensive sinus disease, worse outcomes after surgical therapy for CRSwNP, higher recurrence rates, higher postoperative symptom scores, and less improvement in both disease-specific and general quality of life [30,31]. In this scenario, some authors have tried to establish optimal cutoffs to grade levels of eosinophilia, and classifications have often been achieved by tissue microscopy. Various reports have suggested different threshold values for tissue eosinophilia, ranging from >5 eosinophils/HPF to >120 eosinophils/HPF [32]. Kountakis [33] et al. established that patients with more than five eosinophils/HPF in sinus tissue had a higher frequency of polyps and asthma and higher CT and endoscopy scores than patients without sinus tissue eosinophilia. Soler et al. observed that in CRS patients, the greatest impact on quality-of-life outcomes was seen when the mucosal infiltrate reached >10 eosinophils/HPF [34].

In our study, we confirmed the important predictive value of identifying local eosinophilia and establishing its grade. The two clusters were significantly different in terms of nasal eosinophilic count and patients with a type 2-high inflammatory profile showed a significantly higher eosinophilic count at nasal cytology. On the other hand, the type 2-low group included patients without eosinophils at nasal cytology or with a lower count. Finally, by ROC analyses we observed that an eosinophil count >15.5 eosinophils/hpf was highly predictive of a type 2-high inflammatory profile. We believe that this point should be addressed in future studies and additional research is needed to fully establish the modality and optimal cutoff value that identifies clinically relevant mucosal eosinophilia.

The present study has some limitations that should be briefly discussed. First, several materials may be used to evaluate biomarkers, such as peripheral blood, tissue biopsy, or nasal secretions. Peripheral blood is easier to obtain, but may not always reflect local nasal inflammatory processes and is often a poor proxy for the nasal microenvironment, especially when measuring the load of inflammation [26,27,28]. Analyses in tissue require a more invasive approach by biopsy, requiring more experience; for all these reasons we preferred to investigate cytokines in nasal secretion as in our previous experience. Despite this, some authors have suggested that there may be a somewhat inconsistent correlation between nasal secretion levels and those in serum and nasal polyp tissue [35,36]. Furthermore, we did not measure local levels of IgE and sensitization to bacterial antigens such as *Staphylococcus aureus*; however, most previous studies have assessed the local levels with less emphasis on serum values [37]. Furthermore, the cross-sectional nature of this study considers the cytokine levels in nasal mucus at one time point, although the cytokine levels may change over time according to periods of worsening symptoms/disease, and relative to other periods of stability. Interestingly, we observed that a cut-off of IL-5 was predictive of high type 2 inflammation load; nevertheless, given the relatively small sample size of the studied population, this result needs to be confirmed in larger cohorts. Finally, in the present study, we did not find a difference in the proportion of asthmatic patients and NERD in the two groups. Several studies have demonstrated that asthmatic patients and NERD are predominantly characterized by T2 inflammation. However, the data are conflicting as to whether nasal type 2 cytokine levels in NERD patients are increased or are similar to non-intolerant patients. A future study should clarify this aspect and the data should be verified by large-scale multicenter studies.

## 5. Conclusions

Our research indicates a potential role of type 2 biomarkers obtained noninvasively (i.e., in nasal secretions) in stratifying CRSwNP patients based on the load of inflammation and the possibility of personalizing treatments with surgery, pharmacotherapy and/or innovative biologics. By analyzing type 2 cytokine profiles, we identified two clusters that are significantly different based on the inflammatory load. The first was characterized by high levels of Il-4, Il-5, IL-6, and a high-grade eosinophil count (type 2–high). In the second (type-2 low), lower levels of cytokines were detected in nasal secretions and eosinophilic inflammation was poor or absent. Interestingly, we observed that type 2-high patients showed poorer clinical performance with a substantially higher subjective and objective disease severity as documented by clinical scores such as SNOT-22, LMS, and LKES; in addition, in these patients we observed an increased risk of recurrence of disease following sinus surgery and a greater need for systemic corticosteroids to achieve adequate disease control.

The clinical applicability of our observations and the ease of implementation in clinical practice depends on the standardization of methods to measure biomarkers in nasal secretions, such as those described in this study as well as cost effectiveness. In the future, further investigations should be carried out to identify and validate the best biomarkers and the optimal means of clustering CRS patients to optimize outcomes. Despite all efforts, many patients remain “difficult to treat” and do not respond to surgical treatments. Therefore, there is the need to improve the management strategy of these patients. The most promising results so far include the new biological treatments that are paving the way for a change in the therapeutic approach [10,11,12,13,14,15,16,17,18,19,20,21,22,23,24,25,26,27,28,29,30,31,32,33,34,35,36,37,38].

In the near future, biomarkers can be employed to aid in the prediction of response to specific treatments in integrated care pathways. Therefore, in parallel with ongoing research to better understand the type 2 pathways in CRSwNP, there should be interest in biomarkers to distinguish between a high and low inflammatory load. From a clinical point of view, this aspect may be of particular importance in predicting a poor response to specific treatments such as local/systemic corticosteroids and surgery, and focusing attention on patients who require early treatment with biologics. Hence, it is imperative to cluster CRS patients and to accurately define the severity of the disease to implement integrated care pathways in order to select the optimal treatment and ideal timing from a personalized perspective.

## Figures and Tables

**Table 1:** Clinical characteristics of the studied population.

Epidemiology	
Age (mean ± SD; range)	44.3 ± 15.01; 28–76
Female (n/total; %)	16/44; 36.4%
Male (n/total; %)	28/44; 63.6%
**Phenotyping**	
Concomitant allergy (n/total; %)	20/44; 45.4%
Previous sinonasal surgery	42/44; 95.4%
Number of previous sinonasal surgeries (mean ± SD)	1.75 ± 1.18
Concomitant asthma (n/total; %)	25/44; 56.8%
Peripheral blood hyper-eosinophilia (n/total; %)	14/44; 31.8%
NERD (n/total; %)	14/44; 31.8%
Smoking (n/total; %)	16/44; 36.4%
SNOT-22 (mean ± SD)	47.1 ± 13.71
VAS nasal obstruction (mean ± SD)	7.6 ± 1.96
VAS impaired sense of smell (mean ± SD)	7.8 ± 2.24
LKES (mean ± SD)	13.61 ± 3.02
CT Lund Mackay score (mean ± SD)	17.0 ± 3.69
Number of patients needing >3 brief cycles of OCS in the last year (n/total; %)	18/44; 40.9%

**Table 2:** Levels of cytokines in the two groups. Values are reported as mean ± standard deviation. The right column shows the *p*-value of *t*-test comparison between the two groups.

	Type 2 High (n = 15)	Type 2 Low (n = 29)	*p* Values
IL-5	3.4 ± 2.0 ng/L	1.6 ± 0.4 ng/L	*p* < 0.01
IL-6	4.1 ± 3.3 ng/L	1.3 ± 0.9 ng/L	*p* < 0.05
IL-4	2.2 ± 1.1 ng/L	1.3 ± 0.2 ng/L	*p* < 0.05
IL-33	2.2 ± 2.1 ng/L	1.5 ± 1.4 ng/L	*p* > 0.05
Eosinophil count/hpf	27.8 ± 6.1	4.7 ± 2.5	*p* < 0.05

**Table 3:** Comparison of the most relevant clinical characteristics between the two subgroups of patients (type 2-low and -high). The right column shows the *p*-value of *t*-test comparison between the two groups. Absolute values are intended as mean ± SD.

	Type 2 High (n = 15)	Type 2 Low (n = 29)	*p* Values
Asthma, n/total (%)	10/15 (66.6%)	15/29 (51.17%)	*p* > 0.05
NERD, n/total (%)	3/15 (20%)	6/29 (20.6%)	*p* > 0.05
Allergy, n/total (%)	7/15 (46.6%)	13/29 (44.8%)	*p* > 0.05
Peripheral blood Hypereosinophilia, n/total (%)	8/15 (53.3%)	6/29 (20.6%)	*p* < 0.01
SNOT-22	54.1 ± 10.1	42.7 ± 13.8	*p* < 0.01
VAS nasal obstruction	8.5 ± 1.5	7.0 ± 1.9	*p* < 0.01
VAS impaired sense of smell	8.8 ± 1.7	7.0 ± 2.3	*p* < 0.01
LKES	14.9 ± 2.5	12.8 ± 3.0	*p* < 0.01
CT Lund Mackay score	19.8 ± 1.6	15.2 ± 3.4	*p* < 0.01
Number of previous surgeries	2.3 ± 1.1	1.5 ± 1.1	*p* < 0.05
Number of patients needing >3 brief cycles of OCS in the last year	10/15 (66.6%)	8/29 (27.6%)	*p* < 0.01

## Data Availability

The data presented in this study are available on request from the corresponding author.
